# Tipping Cascades in a Multi-patch System with Noise and Spatial Coupling

**DOI:** 10.1007/s11538-021-00943-y

**Published:** 2021-09-30

**Authors:** Abhishek Mallela, Alan Hastings

**Affiliations:** 1 Department of Mathematics, University of California Davis, Davis, CA 95616 USA; 2 Department of Environmental Science and Policy, University of California Davis, Davis, CA 95616 USA; 3 Santa Fe Institute, Santa Fe, NM 87501 USA

**Keywords:** Tipping points, Stochasticity, Allee effects, Alternative stable states, Resilience, Perturbations

## Abstract

Forecasting tipping points in spatially extended systems is a key area of interest to ecologists. A slowly declining spatially distributed population is an important example of an ecological system that could exhibit a cascade of tipping points. Here, we develop a spatial two-patch model with environmental stochasticity that is slowly forced through population collapse, in the presence of changing environmental conditions. We begin with a basic spatial model, then introduce a fast–slow version of the model using geometric singular perturbation theory, followed by the inclusion of stochasticity. Using the spectral density of the fluctuating subpopulation in each patch, we derive analytic expressions for candidate indicators of population extinction and evaluate their performance through a simulation study. We find that coupling and spatial heterogeneity decrease the magnitude of the proposed indicators in coupled populations relative to isolated populations. Moreover, the degree of coupling dictates the trends in summary statistics. We conclude that this theory may be applied to other contexts, including the control of invasive species.

## Introduction

Complex systems can have thresholds, referred to as tipping points or catastrophic bifurcations, that mark an abrupt shift to an alternate dynamic regime. Such systems have been actively studied across a wide range of fields (Scheffer 2009), including ecology (Hastings and Wysham 2010; Carpenter et al. 2011; Boerlijst et al. 2013; Scheffer et al. 2015; Pace et al. 2015), financial systems (May et al. 2008; Battiston et al. 2016; Tu et al. 2020), climate science (Dakos et al. 2008; Boulton and Lenton 2015; Lenton 2020) and medicine (van de Leemput et al. 2014; Meisel et al. 2015; Maturana et al. 2020). Detecting tipping points and predicting their associated dynamics presents significant challenges, because system observables may show negligible changes until the point of no return is reached. Since a series of tipping points can manifest through domino dynamics, as a unidirectional type of tipping cascade, there is an urgent need to adequately address these challenges. Fortunately, a new research frontier has emerged, harnessing noise as an informer of the sophisticated and often counterintuitive dynamics of interconnected systems.

**Table 1:** Parameter values used for numerical simulations

Parameter	Symbol	Value (per unit time)
Coupling strength (high level of dispersal)	$d_h$	1
Coupling strength (moderate level of dispersal)	$d_m$	0.1
Coupling strength (low level of dispersal)	$d_l$	0.01
Multiplicative noise level	$\sigma _{\mu }$	0.05
Initial value of measure of patch quality (homogeneous model)	$\beta$	0.99
Measure of patch quality (strong source patch 1)	$\beta _1$	0.99
Measure of patch quality (weak source patch 1)	$\beta _1$	0.2
Initial value of measure of patch quality (in deteriorating patch 2)	$\beta _2$	0.99
Rate of change of measure of patch quality (deteriorating patch)	$\beta _0$	0.001
Initial value of population $x_i$ (homogeneous system)	$x_i(0)$	1+$\sqrt{0.99}$
Initial value of population $x_1$ (strong source patch)	$x_1(0)$	1+$\sqrt{0.99}$
Initial value of population $x_1$ (weak source patch)	$x_1(0)$	1+$\sqrt{0.2}$
Initial value of population $x_2$ value (deteriorating patch)	$x_2(0)$	1+$\sqrt{0.99}$

**Table 2:** First derivatives of each statistic, assuming $\beta \in (0,1)$ and $d,\sigma _a, \sigma _{\mu } \in (0,\infty )$

Statistic $h(\beta ,d)$	$\dfrac{\partial }{\partial \beta } h(\beta ,d)$	$\dfrac{\partial }{\partial d} h(\beta ,d)$
$v_{\mu }(\beta ,d)$	$-\frac{\left( 2 \left( \sqrt{\beta }+1\right) ^2 \beta +d^2-2 (\beta -1) \sqrt{\beta } d\right) \sigma _{\mu }^2}{16 \beta ^{3/2} \left( \beta +\sqrt{\beta }+d\right) ^2}$	$-\frac{\left( \sqrt{\beta }+1\right) ^2 \sigma _{\mu }^2}{8 \left( \beta +\sqrt{\beta }+d\right) ^2}$
$\mathrm {CV}_{\mu }(\beta ,d)$	$-\frac{\left( 2 \sqrt{\beta }+1\right) \left( 2 \left( \beta +\sqrt{\beta }\right) +d\right) \left( 2 \left( \sqrt{\beta }+1\right) ^2 \beta +d^2+2 \left( \beta +\sqrt{\beta }\right) d\right) \sigma _{\mu }}{8 \sqrt{2} \beta ^2 \left( \beta +\sqrt{\beta }+d\right) ^3 \left( \frac{\left( \sqrt{\beta }+1\right) \left( 2 \left( \beta +\sqrt{\beta }\right) +d\right) }{\sqrt{\beta } \left( \beta +\sqrt{\beta }+d\right) }\right) {}^{3/2}}$	$-\frac{\left( \sqrt{\beta }+1\right) \sigma _{\mu }}{4 \sqrt{2} \left( \beta +\sqrt{\beta }+d\right) ^2 \sqrt{\left( \sqrt{\beta }+1\right) ^2 \left( \frac{1}{\beta +\sqrt{\beta }}+\frac{1}{\beta +\sqrt{\beta }+d}\right) }}$
$\mathrm {acf}_1(\beta ,d)$	$-\frac{\left( 2 e^{2 d} d^2+d \left( 2 \left( \beta +\sqrt{\beta }\right) +\left( 6 \left( \beta +\sqrt{\beta }\right) +1\right) e^{2 d}-1\right) +4 \left( \beta +\sqrt{\beta }\right) ^2 \left( e^{2 d}+1\right) \right) }{2 \sqrt{\beta } \left( 2 \left( \beta +\sqrt{\beta }\right) +d\right) ^2}$	$-\frac{\left( \beta +\sqrt{\beta }\right) e^{-2 \left( \beta +\sqrt{\beta }+d\right) } \left( \left( 2 \sqrt{\beta }+1\right) ^2-e^{2 d}+2 d\right) }{\left( 2\left( \beta +\sqrt{\beta }\right) +d\right) ^2}$
	$$\times \left( 2 \sqrt{\beta }+1\right) e^{-2 \left( \beta +\sqrt{\beta }+d\right) }$$	

**Fig. 1:**
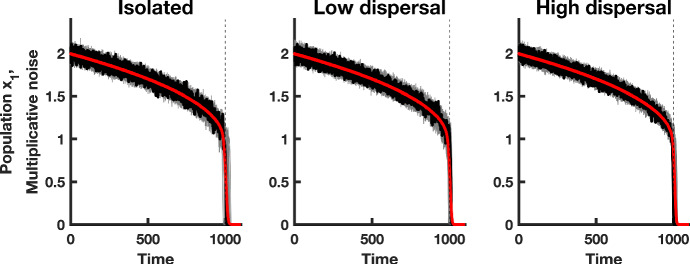
Simulations of population $x_1$ in a homogeneous coupled patch system. The red line shows the mean of 500 realizations of the homogeneous model, a single realization is shown in black, and 50 simulations of the $x_1$ population are shown in gray. The dashed vertical line indicates the time $t^{*}$ at which the saddle-node bifurcation occurs. The first column shows simulations of isolated populations $(d = 0)$, the second column corresponds to simulations of populations coupled through low dispersal levels $(d = 0.01)$, and the last column shows simulations of populations coupled through high dispersal $(d = 1)$. Numerical values for the parameters used in the simulations are provided in Table 1 (Color figure online)

**Fig. 2:**
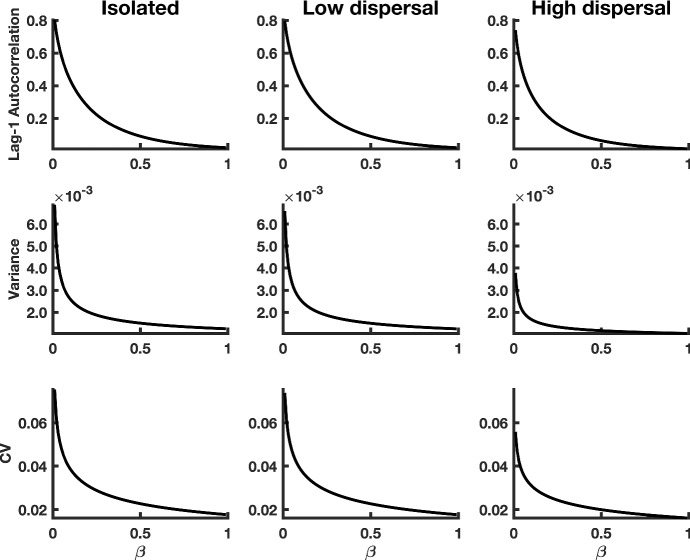
Theoretical predictions for summary statistics of $x_1$ in a homogeneous coupled system. The first column of panels shows summary statistic predictions for isolated patches, the second column of panels displays predictions for $x_1$ populations coupled through low dispersal levels, and the third column of panels corresponds to populations coupled through high dispersal. Parameter values used for the numerical predictions are given in Table 1. Predictions were calculated for fluctuations about the steady state $(1+\sqrt{\beta }, 1+\sqrt{\beta })$ of system (1) (representing the mean of the stochastic fast–slow system) for $\beta$ values ranging from 0.99 down to 0.01, with a spacing of 0.01

**Fig. 3:**
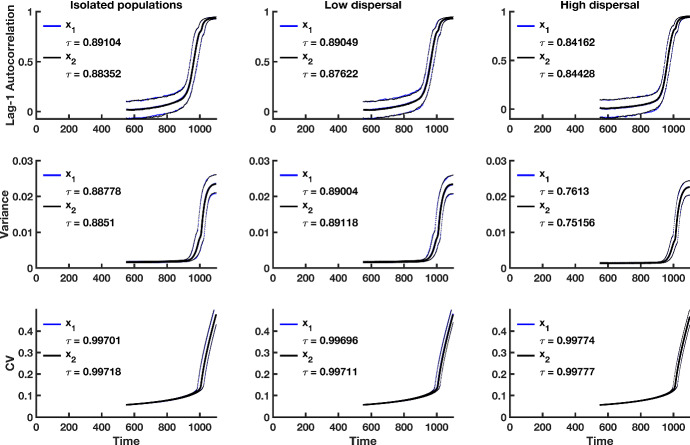
Simulation study predictions for the summary statistics of the $x_1$ population in a homogeneous coupled system with multiplicative noise. Thick blue lines indicate the median value of each statistic for population $x_1$ over 500 simulations; thick black lines correspond to the median value of each statistic for the $x_2$ population over 500 realizations. Dotted lines show the $95\%$ prediction interval for each statistic. The median value of Kendall’s correlation coefficient $\tau$ is reported for each indicator statistic over 500 simulations. The first column of panels is summary statistic predictions for isolated patches, the second column is predictions for populations coupled through low dispersal levels, and the last column shows predictions for populations coupled through high dispersal. Parameter values used for the numerical predictions are given in Table 1 (Color figure online)

**Fig. 4:**
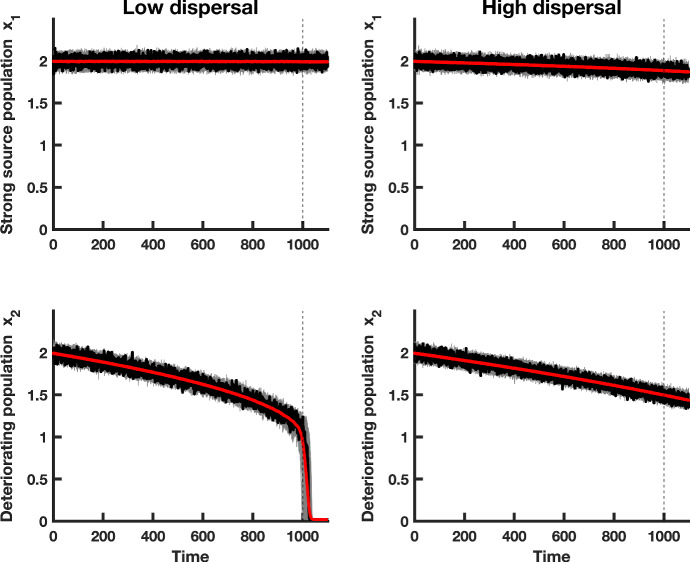
Simulations of both populations, in a heterogeneous coupled patch system with multiplicative noise and a static patch with a good environment (i.e., a “strong source” patch). The red line shows the mean of the $500 \ x_i$ realizations of the heterogeneous model, a single realization is shown in black, and 50 simulations of each subpopulation $x_i$ are shown in gray. The dashed vertical line indicates the time at which the saddle-node bifurcation occurs. The first column of panels displays simulations of populations coupled through low dispersal levels, and the second column corresponds to simulations of populations coupled through high dispersal. Numerical values for the parameters used in the simulations are provided in Table 1 (Color figure online)

A major advancement in this area is the development of early-warning signals (EWS), a suite of statistical tools that are independent of model assumptions or parameterization (Dakos et al. 2012). Instead, they capture generic changes in statistical metrics that occur prior to a bifurcation. The ability to characterize bifurcations is crucial in order to glean insight into upcoming qualitative changes in a system’s behavior. For example, in ecology, populations subject to Allee effects may be described in terms of saddle-node (or fold) bifurcations. Most EWS are rooted in the phenomenon of “critical slowing down," which is a generic property of local bifurcations (Wissel 1984; Strogatz 2001). Akin to a second-order phase transition, critical slowing down (CSD hereafter) results in a longer return time to equilibrium following a perturbation. In ecology, CSD is used as a measure of resilience, the ability of a system to tolerate disturbances and restructure itself while responding to change (Scheffer 2009). In the presence of stochasticity, this manifests as an increase in variance, larger temporal correlations and marked changes in several other statistical measures. Rising variance and lag-1 autocorrelation are commonly used EWS that have been demonstrated in empirical settings (Dakos et al. 2008; Dai et al. 2012; Wouters et al. 2015). These statistics can be derived in linearized models by using stochastic differential equations (Gardiner 2004), but theory for multi-dimensional systems on the verge of tipping has not been clearly elucidated. Moreover, the observability of EWS in real, multi-dimensional networks can be remarkably limited (Boerlijst et al. 2013), underscoring the need for a coherent theory of such systems.

**Fig. 5:**
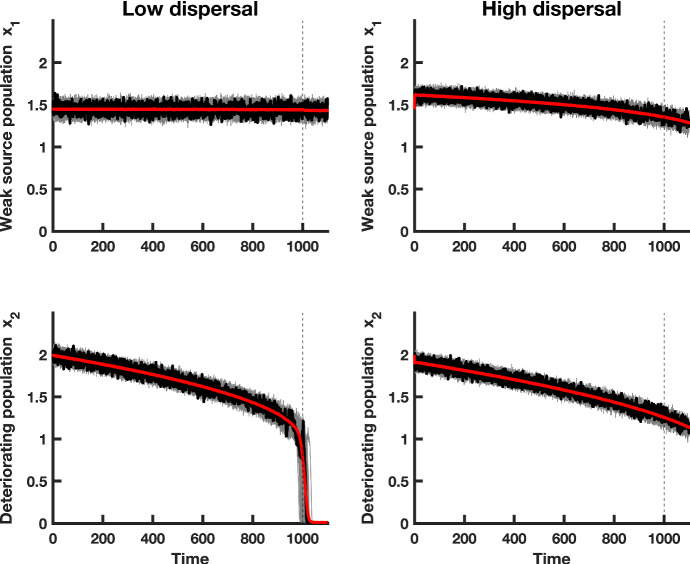
Simulations of the $x_1$ and $x_2$ populations, in a heterogeneous coupled patch system with multiplicative noise and a bad environment (i.e., a “weak source” patch). The red line shows the mean of the $500 \ x_i$ realizations of the heterogeneous model, a single realization is shown in black, and 50 simulations of each subpopulation $x_i$ are shown in gray. A transient is observed before the system relaxes to the moving fast–slow steady state. The dashed vertical line indicates the time at which the saddle-node bifurcation occurs. The first column of panels shows simulations of populations coupled through low dispersal levels, and the second column corresponds to simulations of populations coupled through high dispersal. Numerical values for the parameters used in the simulations are provided in Table 1 (Color figure online)

**Fig. 6:**
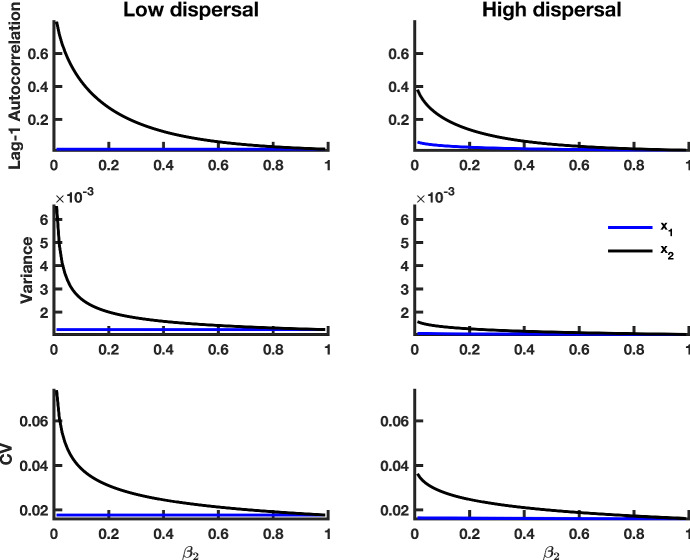
Theoretical predictions for the summary statistics of a heterogeneous coupled system with multiplicative noise and a static patch with a good environment (i.e., a “strong source" patch). The first column shows summary statistic predictions for the $x_1$ and $x_2$ subpopulations coupled through low dispersal levels, and the second column displays predictions for subpopulations coupled through high dispersal. Parameter values used for the numerical predictions are given in Table 1. Predictions were calculated for fluctuations about the steady state $x_1^{*}, x_2^{*}$ of system (1) (representing the mean of the stochastic fast–slow system) for $\beta _2$ values ranging from 0.99 down to 0.01, with a spacing of 0.01, while $\beta _1$ remained constant at 0.99 (Color figure online)

**Fig. 7:**
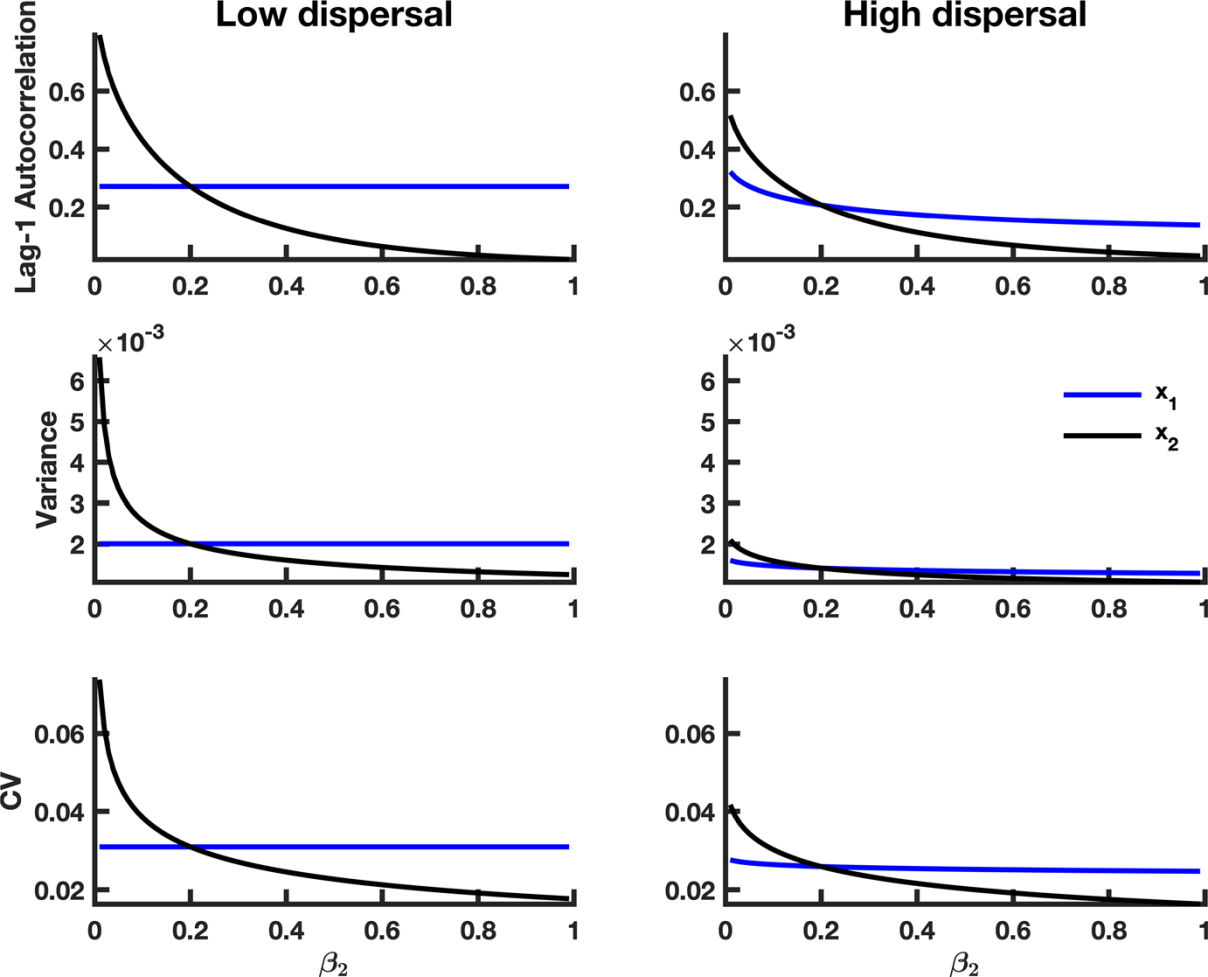
Theoretical predictions for the summary statistics of a heterogeneous coupled system with multiplicative noise and a static patch with a bad environment (i.e., a “weak source" patch). The first column shows summary statistic predictions for the $x_1$ and $x_2$ subpopulations coupled through low dispersal levels, and the second column displays predictions for subpopulations coupled through high dispersal. Parameter values used for the numerical predictions are given in Table 1. Predictions were calculated for fluctuations about the steady state $x_1^{*}, x_2^{*}$ of system (1) (representing the mean of the stochastic fast–slow system) for $\beta _2$ values ranging from 0.99 down to 0.01, with a spacing of 0.01, while $\beta _1$ remained constant at 0.2 (Color figure online)

**Fig. 8:**
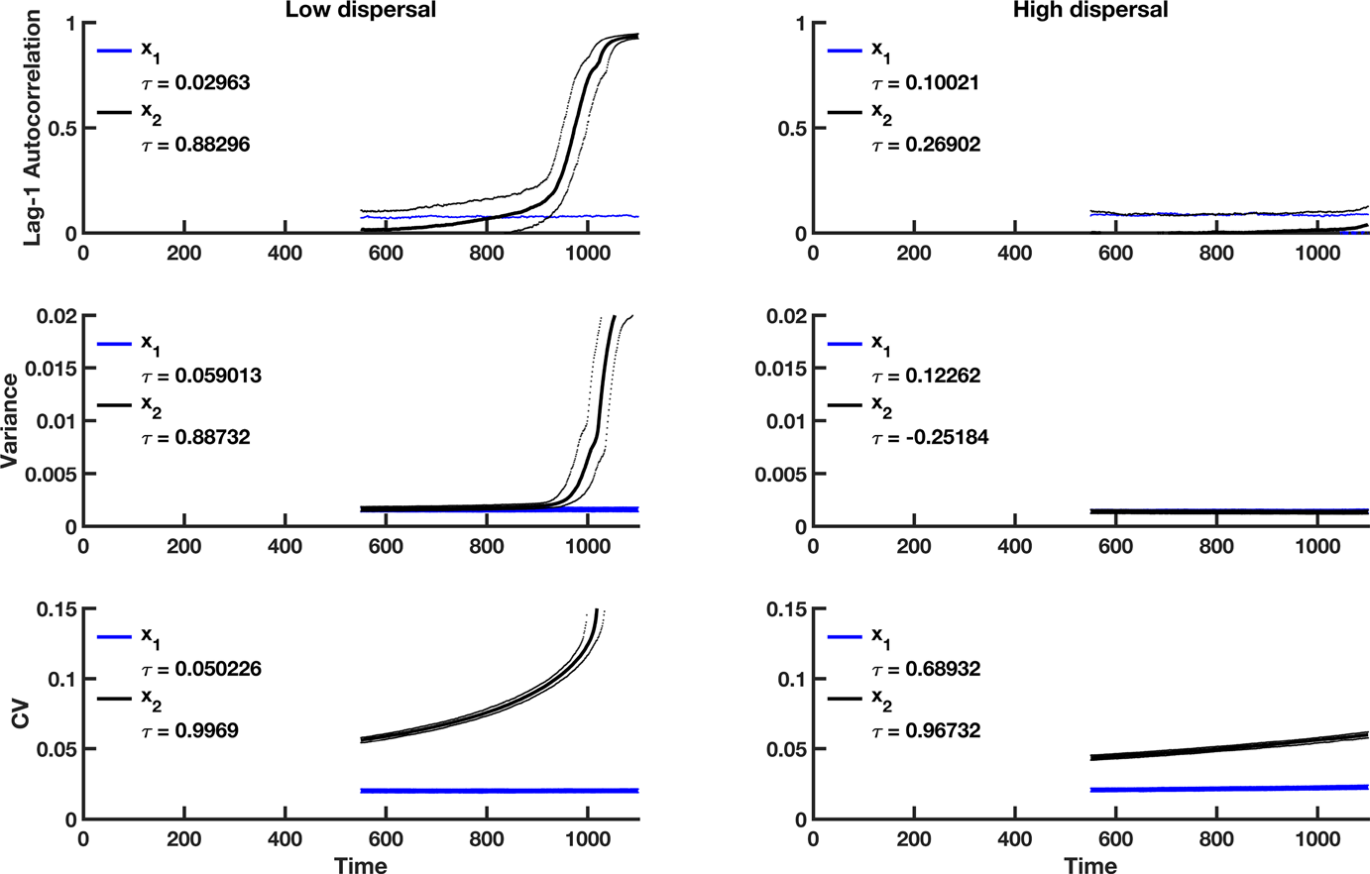
Simulation study predictions for the summary statistics of the $x_1$ **and** $x_2$ population in a heterogeneous coupled system with multiplicative environmental noise and a static patch with a good environment (i.e., a “strong source" patch). Thick blue lines indicate the median value of each statistic for the $x_1$ population over 500 realizations, and thick black lines indicate the median value of each statistic for the $x_2$ population over 500 simulations. Dotted lines correspond to the $95\%$ prediction interval for each statistic. The median value of Kendall’s correlation coefficient $\tau$ is reported for each indicator statistic over 500 simulations. The first column shows predictions for populations coupled through low dispersal levels, and the second column shows predictions for populations coupled through high dispersal. Parameter values used for the numerical predictions are given in Table 1 (Color figure online)

**Fig. 9:**
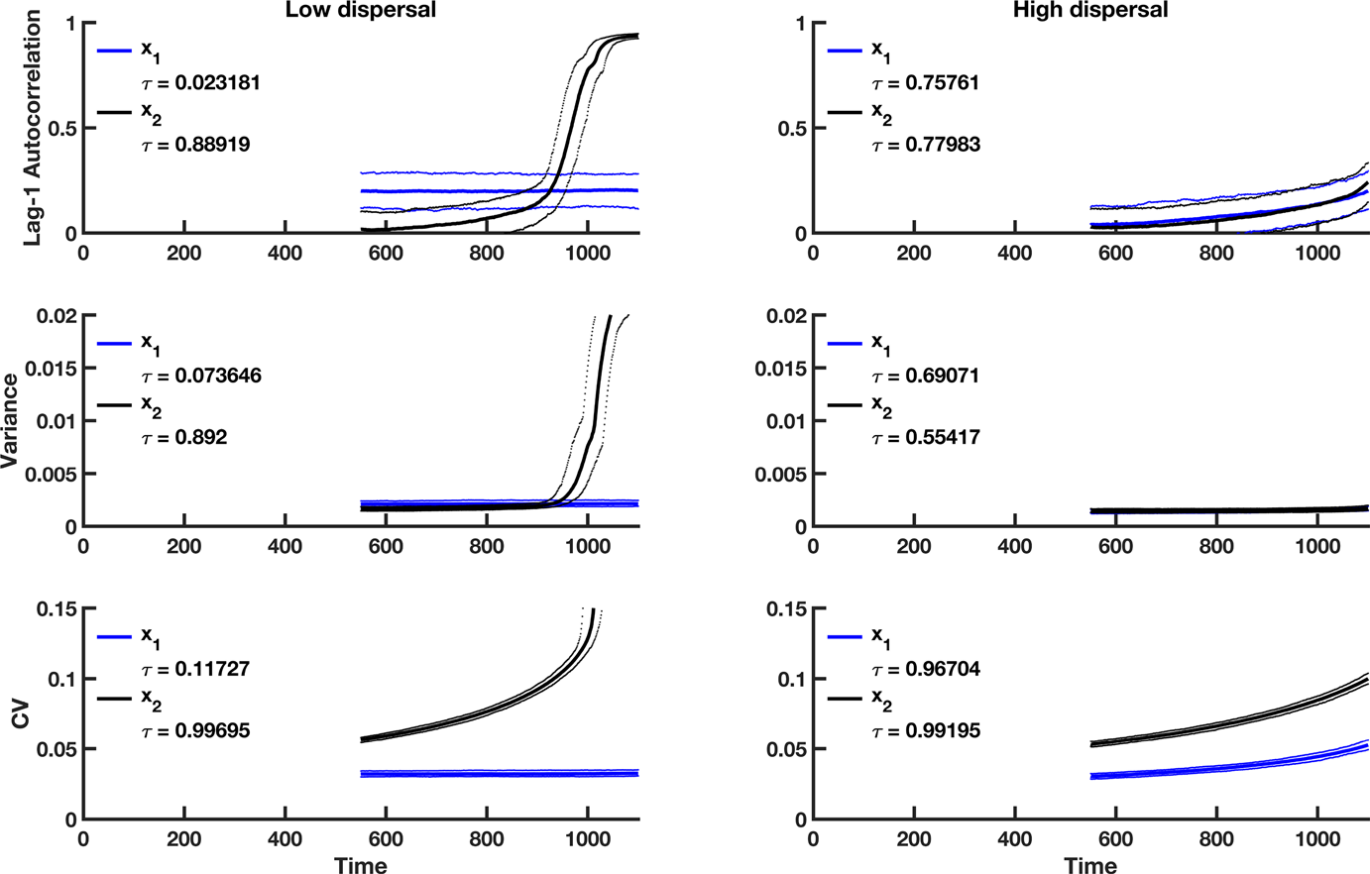
Simulation study predictions for the summary statistics of the $x_1$ and $x_2$ population in a heterogeneous coupled system with multiplicative environmental noise and a static patch with a bad environment (i.e., a “weak source" patch). Thick blue lines indicate the median value of each statistic for the $x_1$ population over 500 realization, and thick black lines indicate the median value of each statistic for the $x_2$ population over 500 simulations. Dotted lines indicate the $95\%$ prediction interval for each statistic. The median value of Kendall’s correlation coefficient $\tau$ is reported for each indicator statistic over 500 simulations. The first column shows predictions for populations coupled through low dispersal levels, and the second column displays predictions for populations coupled through high dispersal. Parameter values used for the numerical predictions are given in Table 1. Initial transient behavior of $x_1$ and $x_2$ (Fig. 5) is captured by the sharp change in statistics over the moving window (Color figure online)

**Fig. 10:**
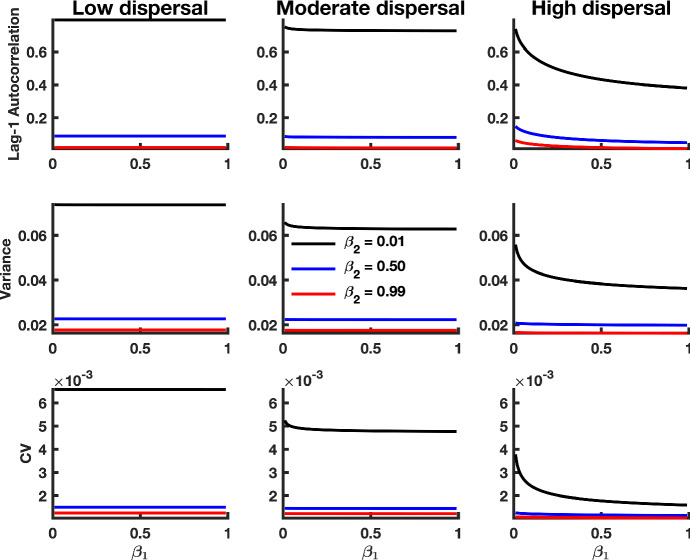
Theoretical predictions for summary statistics of $x_2$ in a tipping cascade with multiplicative noise. The first column of panels shows summary statistic predictions for populations coupled through low dispersal, the second column displays predictions for $x_1$ populations coupled through moderate dispersal, and the third column of panels corresponds to populations coupled through high dispersal. Parameter values used for the numerical predictions are given in Table 1. Predictions were calculated for fluctuations about the steady state $(1+\sqrt{\beta _1}, 1+\sqrt{\beta _2})$ of the spatially heterogeneous system for $\beta _1$ values ranging from 0.99 down to 0.01, with a spacing of 0.01 (Color figure online)

To understand how CSD manifests in a multi-patch system, patch-specific temporal predictors are required. These predictors can also be used to assess whether a single patch can have a clear signal of an impending tipping cascade, or whether every patch needs to be individually monitored. To our knowledge, a predictive theory for temporal statistics of tipping elements that accounts for both stochasticity and coupling is currently lacking. It must be noted, however, that O’Regan made significant headway toward building this theory, although for a metapopulation with logistic growth rates (O’Regan 2018). Thus, the system investigated in that study corresponded to a non-catastrophic, transcritical bifurcation, implying a lack of hysteresis in the dynamics.

Ecological networks are inherently spatial and multi-dimensional, but the influence of space and coupling on tipping points is as yet unclear. Several summary statistics have been proposed as multivariate spatial indicators, such as spatial variance, spatial skewness and spatial kurtosis (Guttal and Jayaprakash 2009; Kéfi et al. 2014). However, spatial statistics are obtained using temporal snapshots of the system under consideration (Carpenter and Brock 2010). For example, the technique of remote sensing may provide higher sensitivity at a lower computational cost than the processing of time series with high frequency. Also, spatial statistics yield information by averaging within patches, which might be less accurate in the presence of patch heterogeneity. In addition, from a theoretical standpoint, spatial statistics are usually difficult to obtain analytically. Thus, temporal indicators that are specific to each patch are necessary to anticipate tipping in connected ecological systems.

To address the aforementioned gap in the literature, we model a metapopulation with Allee effects as a multi-dimensional system in continuous time, where each subpopulation gradually approaches extinction as a result of patch quality evolving over ecological timescales. Gradually degraded metapopulations on the brink of population collapse are an important example of a spatial system that can exhibit a tipping point. Mathematically, a metapopulation that grows locally via Allee effects is characterized by a (codimension-one) saddle-node bifurcation at the tipping point. We address the question of how temporal statistics for subpopulation fluctuations can yield insight into whether or not a metapopulation is losing stability. We extend the framework to explore the effects of spatial heterogeneities on predicting extinction. We also check whether both patches exhibit EWS of tipping or whether partial information (i.e., one patch) is sufficient to inform system management. Finally, we suggest that this theory of Allee effects and stochasticity can be applied to control invasive species, including insect pests (Liebhold et al. 2016).

## Model Description

Models of Allee effects with passive dispersal have been discussed in the literature (Amarasekare 1998; Kang and Lanchier 2011). For an overview, consult Chapter 3 of a classic reference on Allee effects (Courchamp et al. 2008).

### Base Model

We begin our model formulation with the deterministic skeleton for the nondimensionalized model described by Johnson and Hastings, reproduced here for convenience (Johnson and Hastings 2018).

1 $$\begin{aligned} \begin{aligned} \dfrac{\mathrm {d} x_1}{\mathrm {d} t}&= x_1(\beta _1-(x_1-1)^2 )+d(x_2-x_1) \\ \dfrac{\mathrm {d} x_2}{\mathrm {d} t}&= x_2 (\beta _2-(x_2-1)^2 )+d(x_1-x_2) \end{aligned} \end{aligned}$$

In the model above, the parameter $\beta _i$ represents a measure for the quality of the environment by the population denoted by $x_i$. The parameter *d* denotes passive, symmetric diffusion in the system and is a measure of network connectivity. See Johnson’s work for a detailed exposition of the nondimensionalization.

Here, $x_i \ge 0$ for $i = 1,2$ denotes the density of subpopulation *i* that inhabits patch *i*. In the absence of dispersal, subpopulation dynamics are determined by Allee effects at the rate $x_i(\beta _i-(x_i-1)^2)$ in each patch. The model allows for both homogeneity in intrinsic dynamics $(\beta _1 = \beta _2 = \beta )$ and spatial heterogeneity in the environment $(\beta _1 \ne \beta _2)$. The positive steady state of the spatially heterogeneous model $(x_1^{*}, x_2^{*})$ can be obtained numerically.

In the absence of coupling, each population is isolated. If $\beta _i < 0$, population extinction is certain due to the presence of a *fatal* Allee effect. At $\beta _i = 0$, a saddle-node bifurcation occurs. For $0< \beta _i < 1$, each population is bistable with a positive steady state at $x_i = 1+ \sqrt{\beta _i}$ and a stable population extinction state at $x_i = 0$, owing to a *strong* Allee effect. At $\beta _i = 1$, a transcritical bifurcation occurs. Finally, for $\beta _i > 1,$ the extinction state becomes unstable in the regime of the *weak* Allee effect.

In the bistable regime, if environmental conditions are homogeneous (i.e., $0< \beta _1 = \beta _2 = \beta < 1)$ and the subpopulations disperse at a rate $d > 0$, the system has two spatially homogeneous steady states: a positive steady state $(x_1^{*}, x_2^{*}) = (1+\sqrt{\beta },1+\sqrt{\beta })$ and an extinction state at $(x_1^{*}, x_2^{*}) = (0, 0)$. The eigenvalues of the spatially homogeneous system (1) are $-2(\sqrt{\beta }+\beta )$ and $-2(\sqrt{\beta }+\beta + d)$. If the quality of the environment in each patch is degraded, we assume that $\beta$ declines. Therefore, both eigenvalues will decrease in magnitude, and extinction will occur in both patches when the dominant eigenvalue $-2(\sqrt{\beta }+\beta )$ is equal to zero. Hence, critical slowing down prior to population extinction is predicted in the bistable regime of the spatially homogeneous system.

Henceforth, we focus our analyses on the case of the *strong* Allee effect that gives rise to bistability.

### Fast–Slow Model

To study the system’s approach toward a catastrophic collapse, we use a fast–slow model to model the approach to the tipping point. In the spatially homogeneous system, this necessitates that $\beta$ declines slowly relative to the dynamics within each patch. Thus, we modify model (1) to account for a slowly varying quality of environment,

2 $$\begin{aligned} \begin{aligned} \dfrac{\mathrm {d} x_1}{\mathrm {d} t}&= x_1(\beta -(x_1-1)^2 )+d(x_2-x_1) \\ \dfrac{\mathrm {d} x_2}{\mathrm {d} t}&= x_2(\beta -(x_2-1)^2 )+d(x_1-x_2) \\ \dfrac{\mathrm {d} \beta }{\mathrm {d} t}&= -\beta _0 \end{aligned} \end{aligned}$$

where $\beta _0 > 0$ quantifies the rate of change of the parameter $\beta$ in each patch. By Fenichel’s Theorem (Fenichel 1979; Berglund and Gentz 2006; Kuehn 2015), as $\beta _0 \rightarrow 0$, the trajectories of system (2) approach those of the model where $\beta$ remains constant. Since $\beta$ evolves much more slowly than the population dynamics, we assume that $0 < \beta _0 \ll 1,$ and that deteriorating conditions yield a linear decline in $\beta$,

3 $$\begin{aligned} \beta (t) = \beta - \beta _0 t, \end{aligned}$$

with $t^{*} = \beta /\beta _0$ denoting the time at which $\beta (t)$ becomes zero.

In the presence of spatial heterogeneity, the quality of one patch may differ from that of its counterpart. Thus, the underlying growth rates for both patches may be distinct. So, we assume that environmental conditions stay constant in the first patch but that the second patch is slowly degraded, effectively decreasing its population growth rate. This set of assumptions yields the following model:

4 $$\begin{aligned} \begin{aligned} \dfrac{\mathrm {d} x_1}{\mathrm {d} t}&= x_1(\beta _1-(x_1-1)^2 )+d(x_2-x_1) \\ \dfrac{\mathrm {d} x_2}{\mathrm {d} t}&= x_2(\beta _2-(x_2-1)^2 )+d(x_1-x_2) \\ \dfrac{\mathrm {d} \beta _2}{\mathrm {d} t}&= -\beta _0 \end{aligned} \end{aligned}$$

with

5 $$\begin{aligned} \beta _2(t) = \beta _2 - \beta _0 t, \end{aligned}$$

where $t^{*} = \beta _2/\beta _0$ indicates the time that $\beta _2(t)$ becomes zero. By Fenichel’s theorem (Fenichel 1979; Berglund and Gentz 2006; Kuehn 2015), for sufficiently small $\beta _0$, the dynamics of the fast–slow system approach those of the system where $\beta _2$ is fixed at a constant value. Models (2) and (4) can be combined as

6 $$\begin{aligned} \begin{aligned} \dfrac{\mathrm {d} x_1}{\mathrm {d} t}&= x_1(\beta _1(t)-(x_1-1)^2 )+d(x_2-x_1) \\ \dfrac{\mathrm {d} x_2}{\mathrm {d} t}&= x_2(\beta _2(t)-(x_2-1)^2 )+d(x_1-x_2) \end{aligned} \end{aligned}$$

where $\beta _1(t) = \beta _2(t) = \beta (t)$ in the case of spatial homogeneity (see (2) and (3)). In the spatially heterogeneous scenario, we assume that $\beta _1(t) = \beta _1$ and that $\beta _2(t)$ is defined by (5).

### Stochastic Model

We now derive a system of Itô stochastic differential equations (SDEs) that describes spatially homogeneous metapopulations by assuming that exogenous noise can influence patch dynamics.

#### Multiplicative Noise

Following the derivation in other work (O’Regan 2018), and assuming that $\sigma _{\mu }$ is identical for both patches, we obtain the following system of SDEs:

7 $$\begin{aligned} \begin{aligned} \mathrm {d} x_1 = (x_1(\beta _1(t)-(x_1-1)^2 ) - dx_1 + dx_2) \mathrm {d} t + \sigma _{\mu } x_1 \mathrm {d} W_1 \\ \mathrm {d} x_2 = (x_2(\beta _2(t)-(x_2-1)^2 ) - dx_2 + dx_1) \mathrm {d} t + \sigma _{\mu } x_2 \mathrm {d} W_2 \end{aligned} \end{aligned}$$

Since random disturbances scale with the population density $x_i$ in each patch, system (7) describes a model with multiplicative noise. Note that system (7) is a stochastic analogue of the fast–slow system (6).

#### Simulations of the Homogeneous Stochastic Models

To study the behavior of the stochastic fast–slow model as the tipping point is approached, we simulated the model using the parameters in Table 1. Figure 1 shows realizations of the $x_1$ subpopulation in isolated patches $(d = 0)$, as compared to simulations of systems where the $x_1$ population is coupled to another patch, through low and high dispersal, and under homogeneous environmental conditions. Coupling patches through dispersal dampens the environmental fluctuations in each patch, as compared to the case of no dispersal. When coupling is low, and intrinsic dynamics are equal in each patch, coupled populations fluctuate on a similar level as that of isolated populations. When coupling is high, the simulations indicate dampened fluctuations due to the presence of coupling. Finally, it is clear from the simulations that the stochastic fast–slow system evolves more slowly toward extinction than the time $t^{*}$ in which $\beta (t)$ reaches zero.

## Analytic Derivations

In order to predict subpopulation extinction using time series data, we aim to understand the nature of subpopulation fluctuations with temporal leading indicator statistics. In this section, we will show that three indicator statistics change systematically as tipping becomes increasing likely, as a direct consequence of critical slowing down. The steady state here is chosen as the mean of the quasi-stationary population distribution. Consequently, we set $\beta _i(t) = \beta _i$ in model (7) and quantify the behavior of fluctuations in the vicinity of the positive steady state $(x_1^{*}, x_2^{*})$ of model (1). Although the fast–slow model assumes that the mean evolves slowly through time, the steady state is a faithful approximation of the mean of the fast–slow models, provided that the intrinsic growth rate for each patch changes sufficiently slowly.

To derive summary statistics for fluctuations about the positive steady state, we note that we can express system(7) as follows:

8 $$\begin{aligned} \begin{aligned} \mathrm {d} x(t) = f(x(t),t) dt + \sqrt{D(x(t),t)} \mathrm {d} W(t), \end{aligned} \end{aligned}$$

where $x(t) = (x_1, x_2),$ the terms of the mean vector *f*(*x*(*t*), *t*) are $f_i(x(t), t) = x_i(\beta _i(t)-(x_i-1)^2) - dx_i + dx_j$ and the entries of the variance–covariance matrix *D*(*x*(*t*), *t*) are $D_{ii}(x(t), t) = \sigma _{\mu }^2 x_i^2$. The probability distribution *P*(*x*(*t*), *t*) of the solutions of system (8) satisfies the forward Kolmogorov equation (Allen 2010; O’Regan 2018):

9 $$\begin{aligned} \begin{aligned} \dfrac{\partial P(x(t),t)}{\partial t} =&-\sum \limits _{i=1}^2 \dfrac{\partial }{\partial x_i} {[f_i(x(t),t)P(x(t),t)]} \\&+ \dfrac{1}{2} \sum \limits _{i=1}^2 \sum \limits _{j=1}^2 {\dfrac{\partial ^2}{\partial x_i \partial x_j}[D_{ij}(x(t),t) P(x(t),t)]} \end{aligned} \end{aligned}$$

### Model Linearization

To characterize the behavior of fluctuations near the positive steady state, we perform a Taylor expansion of the terms in the mean vector and covariance matrix about $(x_1^{*}, x_2^{*})$ and truncate at leading order:

10 $$\begin{aligned} \begin{aligned} f_i(x_1,x_2,t)&\approx f_i(x_1^{*},x_2^{*},t) + \dfrac{\partial f(x_1^{*},x_2^{*},t)}{\partial x_1} z_1 + \dfrac{\partial f(x_1^{*},x_2^{*},t)}{\partial x_2} z_2 + \ldots \\&\approx 0 + \sum \limits _{j=1}^{2} a_{ij} z_j \ldots , \end{aligned} \end{aligned}$$

where $a_{ij}$ refers to the partial derivatives of $f_i$ and $z_i = x_i - x_i^{*}$ denotes perturbations from the steady state. Similarly,

11 $$\begin{aligned} D_{ij}(x_1,x_2,t) \approx D_{ij}(x_1^{*},x_2^{*},t) + \cdots \end{aligned}$$

The entries $a_{ij}$ of the Jacobian matrix are given by $a_{ii} = \beta _i - d - 1 + 4 x_i^{*} - 3 (x_i^{*})^2$ and $a_{ij} = d$, for $i \ne j,$ and the terms of the variance–covariance matrix are $D_{ii} = \sigma _{\mu }^2 (x_i^{*})^2$. The joint probability distribution of fluctuations $z(t) = (z_1, z_2)$ from the steady state satisfies

12 $$\begin{aligned} \begin{aligned} \dfrac{\partial \varPi (z(t),t)}{\partial t} =&-\sum \limits _{i=1}^2 \dfrac{\partial }{\partial z_i} \varPi (z(t),t)) \left( \sum \limits _{j=1}^{2} a_{ij}z_i \right) \\&+ \dfrac{1}{2} \sum \limits _{i=1}^2 \sum \limits _{j=1}^2 {\dfrac{\partial ^2}{\partial z_i \partial z_j}[D_{ij}\varPi (z(t),t)]} \end{aligned} \end{aligned}$$

Solutions of the following system of stochastic differential equations:

13 $$\begin{aligned} \begin{aligned} \mathrm {d} z_1&= (a_{11}z_1 + a_{12}z_2) \mathrm {d} t + \sqrt{D_{11}} \mathrm {d} W_1 \\ \mathrm {d} z_2&= (a_{21}z_1 + a_{22}z_2) \mathrm {d} t + \sqrt{D_{22}} \mathrm {d} W_2 \end{aligned} \end{aligned}$$

share the same probability distribution $\varPi (z(t),t)$ (Allen et al. 2008; O’Regan 2018).

### Spectral Density

To derive leading indicators of extinction, we begin with the spectral density of the fluctuating subpopulation within each patch. The technique of Fourier transformation can be used to obtain this function. For the full derivation, we refer the interested reader to prior work (Nisbet and Gurney 1982; O’Regan 2018).

Briefly, we note that any continuous function *z*(*t*) defined for $-L/2 \le t \le L/2$ may be expressed in terms of its Fourier transform ${\widetilde{z}}(\omega )$,

14 $$\begin{aligned} \begin{aligned} z(t) = \dfrac{1}{2 \pi } \int _{-\infty }^{\infty } {\widetilde{z}}(\omega ) \exp (i \omega t)~ \mathrm {d}\omega , \end{aligned} \end{aligned}$$

with $\omega$ denoting angular frequency. The Fourier transform of *z*(*t*) is then given by

15 $$\begin{aligned} \begin{aligned} {\widetilde{z}}(\omega ) = \int _{-L/2}^{L/2} z(t) \exp (-i \omega t)~ \mathrm {d}t. \end{aligned} \end{aligned}$$

We rewrite system (13) in a form that lends itself to the method of Fourier transformation:

16 $$\begin{aligned} \begin{aligned} \dfrac{\mathrm {d} z_1}{ \mathrm {d} t} = a_{11}z_1(t) + a_{12} z_2(t) + \sqrt{D_{11}} \varGamma _1(t), \\ \dfrac{\mathrm {d} z_2}{ \mathrm {d} t} = a_{21}z_1(t) + a_{22} z_2(t) + \sqrt{D_{22}} \varGamma _2(t), \end{aligned} \end{aligned}$$

where $\varGamma _1(t)$ and $\varGamma _2(t)$ denote white noise processes associated with the covariance matrix $\{D_{ij}\}$. Fourier transformation of system (16) yields:

17 $$\begin{aligned} \begin{aligned} i\omega {\widetilde{z}}_1 (\omega ) = a_{11} {\widetilde{z}}_1 (\omega ) + a_{12} {\widetilde{z}}_2 (\omega ) + \sqrt{D_{11}} {\widetilde{\varGamma }}_1(\omega ), \\ i\omega {\widetilde{z}}_2 (\omega ) = a_{21} {\widetilde{z}}_1 (\omega ) + a_{22} {\widetilde{z}}_2 (\omega ) + \sqrt{D_{22}} {\widetilde{\varGamma }}_2(\omega ),\end{aligned} \end{aligned}$$

where ${\widetilde{z}}_1 (\omega ), {\widetilde{z}}_2 (\omega ), {\widetilde{\varGamma }}_1 (\omega )$ and ${\widetilde{\varGamma }}_2 (\omega )$ are the Fourier transforms of the functions $z_1(t), z_2(t), \varGamma _1(t)$ and $\varGamma _2(t)$, respectively. We can then obtain ${\widetilde{z}}_1(\omega )$ as

18 $$\begin{aligned} \begin{aligned} {\widetilde{z}}_1(\omega ) = \dfrac{(a_{22} - i \omega ) \sqrt{D_{11}} \varGamma _1(\omega )}{\delta -\omega ^2 - i T \omega } - \dfrac{a_{12} \sqrt{D_{22}} \varGamma _2(\omega )}{\delta -\omega ^2 - i T \omega }, \end{aligned} \end{aligned}$$

where *T* and $\delta$ are the trace and determinant of the Jacobian matrix $\{a_{ij}\}$, respectively. Using (18), we can establish the spectral density of the fluctuations, which we denote by

19 $$\begin{aligned} \begin{aligned} S_1 (\omega ) = \dfrac{D_{11} a_{22}^2 + D_{22} a_{12}^2 + D_{11} \omega ^2}{(\omega ^2 - \delta )^2 + T^2 \omega ^2}. \end{aligned} \end{aligned}$$

(See the Appendix in the work by O’Regan for the complete derivation.) Similarly, the spectral density of fluctuations of the subpopulation in the second patch can be obtained as:

20 $$\begin{aligned} \begin{aligned} S_2 (\omega ) = \dfrac{D_{22} a_{11}^2 + D_{11} a_{21}^2 + D_{22} \omega ^2}{(\omega ^2 - \delta )^2 + T^2 \omega ^2}. \end{aligned} \end{aligned}$$

### Leading Indicators in the Spatially Homogeneous Setting

Here, we derive analytic expressions for the variance, coefficient of variation, lag-1 autocovariance function and lag-1 autocorrelation function for the spatially homogeneous model with $0< \beta < 1$ and $d > 0$. For the spatially homogeneous model, $a_{11} = a_{22} = -2\sqrt{\beta }(\sqrt{\beta }+1) - d$ and $a_{12} = a_{21} = d$. The spectral density of the fluctuations of the subpopulations in each patch *i* is given by

21 $$\begin{aligned} S_i(\omega ) = \dfrac{\sigma _{\mu }^2 (1+\sqrt{\beta })^2 [2 \sqrt{\beta }(\sqrt{\beta } + 1) + d]^2 + \sigma _a^2 d^2 + \sigma _a^2 \omega ^2}{(\omega ^2 - [(2 \sqrt{\beta } (\sqrt{\beta } + 1) + d)^2 - d^2])^2 + 4(2 \sqrt{\beta }(\sqrt{\beta }+1) + d)^2 \omega ^2} \end{aligned}$$

To obtain the variance of the fluctuations, we integrate the spectral density over all frequencies:

22 $$\begin{aligned} \dfrac{1}{2\pi } \int _{-\infty }^{\infty } S_i(\omega ) d \omega = \dfrac{1}{\pi } \int _{0}^{\infty } S_i(\omega ) \mathrm{d}\omega . \end{aligned}$$

Evaluating this integral expression yields

23 $$\begin{aligned} v_{\mu }(\beta ,d) = \dfrac{(\sqrt{\beta } + 1)[2 \sqrt{\beta } (\sqrt{\beta } + 1) + d] \sigma _{\mu }^2}{8 \sqrt{\beta }[d + \sqrt{\beta }(\sqrt{\beta }+1)]} \end{aligned}$$

To obtain the autocovariance function, we compute

24 $$\begin{aligned} \dfrac{1}{2\pi } \int _{-\infty }^{\infty } S_i(\omega ) \cos (\omega \tau ) d \omega = \dfrac{1}{\pi } \int _{0}^{\infty } S_i(\omega ) \cos (\omega \tau ) \mathrm{d}\omega \end{aligned}$$

using the evenness of $S_i(\omega ).$ Integrating expression (24) with $\tau = 1$ gives

25 $$\begin{aligned} \begin{aligned} a_{\mu }(\beta ,d)&= \dfrac{\sigma _{\mu }^2 (\sqrt{\beta }+1) [d \exp (2d) + \sqrt{\beta }+ \exp (2d) \sqrt{\beta } + \beta + \exp (2d) \beta ]}{8 \sqrt{\beta }[d + \sqrt{\beta }(\sqrt{\beta }+1)]} \\&\quad \times \exp (-2[d+\sqrt{\beta }(\sqrt{\beta }+1)]) \end{aligned} \end{aligned}$$

Dividing this expression by the variance gives the lag-1 autocorrelation function:

26 $$\begin{aligned} \begin{aligned} acf_1(\beta )&= \dfrac{d \exp (2d) + \sqrt{\beta }+ \exp (2d) \sqrt{\beta } + \beta + \exp (2d) \beta }{d + 2\sqrt{\beta }(\sqrt{\beta }+1)} \\&\quad \times \exp (-2[d+\sqrt{\beta }(\sqrt{\beta }+1)]) \end{aligned} \end{aligned}$$

It can be shown that the variance and lag-1 autocorrelation functions can be written in terms of the eigenvalues $\lambda _1$ and $\lambda _2$ of the spatially homogeneous system:

27 $$\begin{aligned} v_{\mu }(\beta ,d)= & {} \dfrac{\sigma _{\mu }^2}{16}(|\lambda _1| + |\lambda _2|)\dfrac{|\lambda _1|^2}{|\lambda _2|}\left( \dfrac{1}{1 + \sqrt{2 |\lambda _1| + 1}}\right) . \end{aligned}$$

28 $$\begin{aligned} \begin{aligned} acf_1(\beta )=&{} \dfrac{1}{|\lambda _1| + |\lambda _2|}{\left[ |\lambda _1| \exp (-|\lambda _2|) + |\lambda _2| \exp (-|\lambda _1|)\right] } \end{aligned}\end{aligned}$$

To find the coefficient of variation statistic for each patch, we divide the standard deviation of the fluctuations by the subpopulation mean $1+\sqrt{\beta }$ in each patch:

29 $$\begin{aligned} CV_{\mu }(\beta ,d) = \dfrac{\sigma _{\mu } \sqrt{2}}{4 ( 1+\sqrt{\beta })} \sqrt{\dfrac{(1+\sqrt{\beta })(d+2\sqrt{\beta }+2\beta )}{\sqrt{\beta }(d+\beta +\sqrt{\beta })}} \end{aligned}$$

In summary, measures of variability depend on the strength of noise. It can be seen that the leading indicators are continuous functions of $\beta$ and the dispersal parameter *d*. All leading indicator functions exist for $0< \beta < 1$ and are defined for $d > 0$.

### Theoretical Predictions

Next, we are interested in the qualitative behavior of the leading indicators as patch quality is degraded, that is, as the intrinsic patch quality $\beta$ of each subpopulation approaches zero from the right, for $\beta \in (0,1)$. Taking the limit of each expression as $\beta \rightarrow 0^{+}$ yields:

30 $$\begin{aligned}&\lim \limits _{\beta \rightarrow 0^{+}} v_{\mu }(\beta ,d) = +\infty , \end{aligned}$$

31 $$\begin{aligned}&\lim \limits _{\beta \rightarrow 0^{+}} CV_{\mu }(\beta ,d) = + \infty , \end{aligned}$$

32 $$\begin{aligned}&\lim \limits _{\beta \rightarrow 0^{+}} acf_1(\beta ,d) = 1. \end{aligned}$$

To better intuit the behavior of the statistics as the tipping point of the system is approached due to changes in intrinsic dynamics, we compute the first derivative of each statistic with respect to $\beta$ (Table 2). By calculating the first derivative of each function with respect to $\beta$, provided $d > 0$ and $0< \beta < 1$, we find that $v_a(\beta ,d), v_{\mu }(\beta ,d), CV_a(\beta ,d)$, $CV_{\mu }(\beta ,d)$ and $acf_1(\beta ,d)$ are strictly decreasing functions of $\beta$; therefore, all of these functions increase monotonically as $\beta$ approaches zero from the right (Table 2). Thus, we predict strictly increasing trends in lag-1 autocorrelation, variance and coefficient of variation, as extinction is approached in each patch.

To understand the effect of coupling on the behavior of the temporal leading indicators, we examine these functions as *d* approaches zero from above and as *d* approaches positive infinity.

As *d* decreases to zero from above, the limit of each statistic approaches the expression for the statistic in the case without dispersal:

33 $$\begin{aligned}&\lim \limits _{d \rightarrow 0^{+}} v_{\mu }(\beta ,d) = \dfrac{\sigma _{\mu }^2(\sqrt{\beta }+1)}{4 \sqrt{\beta }}, \end{aligned}$$

34 $$\begin{aligned}&\lim \limits _{d \rightarrow 0^{+}} CV_{\mu }(\beta ,d) = \dfrac{\sigma _{\mu }}{2\sqrt{\sqrt{\beta }+\beta }}, \end{aligned}$$

35 $$\begin{aligned}&\lim \limits _{d \rightarrow 0^{+}} acf_1(\beta ,d) = \exp \left[ -2\sqrt{\sqrt{\beta }+\beta }\right] . \end{aligned}$$

Consequently, the summary statistics capture the behavior of the whole system as being similar to that of isolated subsystems. If coupling increases to infinity, the limits are

36 $$\begin{aligned} \lim \limits _{d \rightarrow \infty } v_{\mu }(\beta ,d)= & {} \dfrac{\sigma _{\mu }^2(\sqrt{\beta } + 1)}{8 \sqrt{\beta }}, \end{aligned}$$

37 $$\begin{aligned} \lim \limits _{d \rightarrow \infty } CV_{\mu }(\beta ,d)= & {} \dfrac{\sigma _{\mu } \sqrt{2}}{4\sqrt{\sqrt{\beta }+\beta }}, \end{aligned}$$

38 $$\begin{aligned} \lim \limits _{d \rightarrow \infty } acf_1(\beta ,d)= & {} \exp \left[ -2\sqrt{\sqrt{\beta }+\beta }\right] . \end{aligned}$$

Increasing the degree of patch connectivity muffles the temporal signals that quantify variability. As $d \rightarrow \infty$, the variance approaches 1/2 of the variance in the absence of dispersal. So, in a very well-mixed metapopulation, the temporal variance in each patch will be muted relative to isolated patches. Similarly, as *d* approaches infinity, the coefficient of variation approaches $1/\sqrt{2}$ of its analogue in the absence of coupling. Table 2 shows that the derivative of each function monotonically decreases with *d*, provided $d>0, 0< \beta < 1$ and $\sigma > 0.$

Notice that the lag-1 autocorrelation function approaches $\exp [-2\sqrt{\sqrt{\beta }+\beta }]$ as $\beta$ approaches either 0 or $\infty$. The first derivative of $acf_1(\beta , d)$ with respect to *d* is

$$\begin{aligned} \dfrac{\partial }{\partial d} acf_1(\beta ,d) = -\dfrac{\exp [-2(d+\sqrt{\beta }+\beta )](\sqrt{\beta }+\beta )[2d - \exp (2d) + (1+2\sqrt{\beta })^2]}{(d+2(\sqrt{\beta }+\beta ))^2} \end{aligned}$$

Provided that *d* is strictly positive and $\beta \in (0,1)$, there is a critical point of $acf_1(\beta ,d)$ at

39 $$\begin{aligned} d_c = -\frac{1}{2}(1+2\sqrt{\beta })^2 {-\frac{1}{2}}\mathrm {ProductLog}[-\exp [-(1+2\sqrt{\beta })^2]. \end{aligned}$$

Applying the second derivative test shows that a local minimum of $acf_1(\beta , d)$ occurs at $d_c$:

$$\begin{aligned} \begin{aligned} \dfrac{\partial ^2 acf_1(\beta ,d_c)}{\partial ^2 d}&= \dfrac{8(\sqrt{\beta }+\beta )\exp [-2(\sqrt{\beta }+\beta )]}{\mathrm {ProductLog}[-\exp [-(1+2\sqrt{\beta })^2]]} \\&\quad \times \dfrac{1}{(1+\mathrm {ProductLog}[-\exp [-(1+2\sqrt{\beta })^2]])} \end{aligned} \end{aligned}$$

The second derivative is always positive at $d_c$, because the denominator of the expression above is strictly positive, owing to the dominance of the square term.

As a result, with low dispersal in the spatially homogeneous system, the neighboring points $x_i(t)$ and $x_i(t + 1)$ in a stationary time series are highly correlated. As dispersal increases, the temporal correlation between $x_i(t)$ and $x_i(t + 1)$ decreases, due to mixing between both patches. For $d > d_c$, however, autocorrelation increases because dispersal is sufficiently high to result in a single population. In summary, $x_i(t)$ and $x_i(t + 1)$ are least correlated at intermediate levels of patch connectivity. For intermediate levels of dispersal, we can expect the lag-1 autocorrelation to be lower relative to that of isolated patches, but if coupling is sufficiently high, the lag-1 autocorrelation approaches that of a single patch.

### Numerical Predictions for the Summary Statistics

Numerically evaluating the summary statistics at the mean $(x_1^{*}, x_2^{*}) = (1+\sqrt{\beta }, 1+\sqrt{\beta })$ confirms our theoretical predictions. As $\beta$ approaches zero from the right in each patch, the leading indicators change as predicted by the theory (Fig. 2). The presence of coupling dampens patterns in indicator statistics that measure variability. Furthermore, a high dispersal rate leads to larger changes in the lag-1 autocorrelation statistic for populations in synchrony, in contrast with isolated patches, as predicted with a decrease in $\beta$.

## Simulation Methodology

The theoretical predictions for the summary statistics in Sect. 3 are calculated about the steady state (i.e., the subpopulation mean $(x_1^{*}, x_2^{*}) = (1+\sqrt{\beta }, 1+\sqrt{\beta }))$. In order to test the robustness of the theoretical predictions for the leading indicator statistics under worsening patch quality, we conducted a simulation study using the fast–slow model derived in Sect. 2. Using the parameters in Table 1, we simulated the stochastic fast–slow models approaching a tipping point under low and high dispersal regimes, and under both spatially homogeneous (system (2) and (3)) and heterogeneous (system (4) and (5)) environmental conditions.

Here, we use the simulation procedure implemented in prior work (O’Regan 2018). All computations were performed in MATLAB (MATLAB 2020).

## Results

Increases in lag-1 autocorrelation, variance and coefficient of variation are seen in the simulation study of the coupled two-patch model (Fig. 3). As the theory shows, higher dispersal levels result in decreases in the magnitude and rate of change of the trends in the indicator statistics. Median Kendall correlation coefficient values are all positive, demonstrating that on average, positive trends in indicator statistics occur as tipping is approached. Also, the median correlation coefficient values for the coefficient of variation are very close to 1, indicating that under multiplicative noise, one can expect a strong positive relationship between a worsening environment and the coefficient of variation. This finding is common across dispersal levels. In summary, indicator statistics behave as predicted by the theory, with the coefficient of variation performing consistently well as an indicator of CSD across all cases explored here.

### The Impact of Spatial Heterogeneity on CSD

Spatial systems are generally heterogeneous in nature (Levin 1976; O’Regan 2018). To investigate how spatial heterogeneities affect CSD in a spatially extended dynamical system, and whether the predictions for the leading indicator statistics for the spatially homogeneous model are robust to heterogeneities, we formulated a fast–slow spatially heterogeneous model with multiplicative noise. We assumed that environmental conditions remain constant in the first patch, while conditions steadily decline in the second patch. We investigated the dynamics when patch 1 is a “strong source," meaning that the patch quality is high and population growth occurs under favorable conditions. We also explored the onset of CSD when the first patch is a “weak source," that is, when conditions for population growth are poor, and the population is close to extinction. Figure 4 shows simulations of the fast–slow model with a strong source patch and a deteriorating patch under low and high dispersal. Due to mixing between the subpopulations, both patches decline in habitat quality. Although the second patch deteriorates at the same rate as the declining patches in the spatially homogeneous system, the strong source patch exhibits a rescue effect in the regime of high dispersal. Both patches decline at similar rates due to the high level of connectivity between the subpopulations.

Similar patterns of subpopulation decline due to high levels of coupling are also observed when the first patch is depleted of resources (Fig. 5). When a poor quality patch is coupled with a degrading subpopulation, both subpopulations decline toward extinction and exhibit larger fluctuations compared to subpopulations where one patch has a good environment (compare Figs. 4, 5). The weak source patch does not show a strong rescue effect, even for higher levels of coupling. The spatially heterogeneous metapopulation with a weak source patch appears to be more responsive to the intrinsic dynamics within each patch, because each patch has an initial transient before the system relaxes to the moving steady state of the fast–slow system (Fig. 5). Initially, the subpopulation in patch 1 is buffered from extinction due to dispersal of individuals from patch 2, which has a better initial environmental quality than the second patch.

Figures 4 and 5 suggest the following hypothesis: due to a rescue effect, temporal indicators of CSD should be weaker in a spatially heterogeneous system with a strong source patch than a spatially heterogeneous system with a weak source patch. The latter system is closer to the extinction threshold, whereas the metapopulation with a strong source patch is buffered from extinction.

#### Theoretical Predictions

In order to examine the behavior of leading indicators of extinction in a spatially heterogeneous system, we numerically integrated equations (19) and (20) for each steady-state value $(x_1^{*}, x_2^{*})$ of system (1) (which we used to represent the mean of the stochastic fast–slow system) for $\beta _2$ values ranging between 1 and $10^{-2}$, while $\beta _1$ remained constant (Table 1), and we used the integrals to calculate summary statistics. Figures 6 and 7 show the behaviors of the summary statistics as $\beta _2$ decreases toward zero from the right. In a similar manner as the spatially homogeneous system , the lag-1 autocorrelation function, variance and coefficient of variation all increase, in both patches, as environmental conditions in patch 2 decline. Moreover, trends in leading indicators are dampened with increasing dispersal, just as predicted in the case of the spatially homogeneous system.

Since conditions in the second patch are deteriorating, it is reasonable to expect that subpopulation $x_2$ would exhibit a stronger sign of CSD than the $x_1$ subpopulation subject to a constant growth rate in the first patch. We find that this is indeed the case. Under low dispersal and good conditions in patch 1, $x_2$ exhibits a stronger signal of CSD than $x_1$, as indicated by the magnitude and slope of the summary statistics; this is true under high dispersal as well (Fig. 7).

Under high dispersal and poor environmental quality for the second patch, patterns in leading indicators are similar in both patches (Fig. 7). When dispersal is low, leading indicators obtained from population fluctuations in patch 2 change more rapidly than those obtained from patch 1 as $\beta _2$ approaches zero from the right. Further away from the extinction point at $\beta _2 = 0$, patch 1 has larger variance, lag-1 autocorrelation and coefficient of variation due to poor conditions that make the $x_1$ subpopulation more susceptible to extinction. All of the summary statistics increase, in both patches, as $\beta _2$ approaches zero.

Comparing the magnitudes of the signals in Figs. 6 and 7, we observe stronger signals of CSD in the summary statistics obtained from the spatially heterogeneous model with a weak source patch than the model with a good quality patch. These predictions suggest that the spatially heterogeneous system with a weak source patch, which is near extinction, should exhibit stronger signals of CSD than the model with a good source patch that favors system longevity.

#### Simulation Study Predictions

Predictions for the summary statistics calculated from the spatially heterogeneous fast–slow model over a moving window (Figs. 7, 8) confirm the predicted trends obtained by numerical integration (Figs. 5, 6). From the median $\tau$ values, there is considerably more variability in leading indicator trends than those obtained from simulations of the spatially homogeneous system (compare the median Kendall’s $\tau$ values in Fig. 4 with those from Figs. 8 and 9). Furthermore, the prediction that stronger signals of CSD are observed in the spatially heterogeneous system with a weak source patch than the corresponding system with a strong source patch is robust in the simulated models (compare $\tau$ values in Fig. 8 with those in Fig. 9). Just as in the spatially homogeneous system, the coefficient of variation appears to be the most reliable indicator of extinction.

### Partial Observability in Tipping Cascades

As discussed in Sect. 1, a given multi-dimensional system is likely to be observable in only one dimension. We can thus explore the likelihood of a tipping cascade as follows. By defining the extinction threshold by $L := L_1 = L_2 = 0$, the Allee threshold as $A_i := 1 - \sqrt{\beta _i}$ (Johnson and Hastings 2018) and the high threshold as $H_i := 1 + \sqrt{\beta _i}$ for $i = 1,2$, we notice that $L_i< A_i < H_i$ in the bistable regime of the strong Allee effect. Hence, we can consider the question of whether there exists a signal for CSD in the system, given that only one patch is observable and deteriorates in quality. This amounts to checking whether or not a transition from $H_1$ to $L_1$ in the first patch is captured by the full system, with the second patch maintained at either $H_2$ or $L_2$. In other words, the cases here correspond to $(H_1,H_2) \rightarrow (L_1,H_2)$ and $(H_1,L_2) \rightarrow (L_1,L_2)$, respectively. Together, these two cases capture the scenario of a tipping cascade, whereby the system can begin at a high-high state and end in a catastrophic collapse at the low-low state (note that the states $(L_1,H_2)$ and $(H_1,L_2)$ can be considered as equivalent (Mallela and Hastings 2021)).

In the spatially homogeneous scenario (i.e., $\beta _1 = \beta _2 = \beta$), we note that the thresholds discussed above are the same for both patches. The expressions for the respective leading indicators are also identical across patches. Thus, we can refer to expressions (30) to (32) in Sect. 3, to understand that all of the leading indicators describing CSD in the second patch have strictly increasing trends as extinction is approached in the first patch. Hence, observing the second patch adequately informs our assessment of the first patch.

For purposes of display, we analyze the case of multiplicative noise in the spatially heterogeneous case with $\beta _1 \ne \beta _2$ (Fig. 10). Increases in lag-1 autocorrelation, variance and coefficient of variation for the second patch are seen in a study of the coupled two-patch model with multiplicative noise. Lower values of $\beta _2$, reflecting a poorer quality of the second patch, result in stronger signals of CSD for the first patch. This is true across all dispersal levels. For a fixed combination of $\beta _1$ and $\beta _2$, the strength (magnitude) of the signal decreases with higher dispersal, for both the variance and coefficient of variation. Interestingly, however, in the case of the lag-1 autocorrelation indicator, the signal strength does not decay with increasing dispersal. We note that our analyses here capture both parts of a tipping cascade. In summary, the lag-1 autocorrelation statistic appears to be robust with respect to partial observability of tipping throughout the system.

## Discussion

Predicting tipping cascades is a complex task. Temporal, patch-specific indicators are potentially useful for anticipating catastrophic events in networks with poor connectivity and environmental noise. We formulated a stochastic fast–slow two-patch model that is valid for different environmental conditions. By simulating the stochastic fast–slow model, we showed that predicted trends in the leading indicators are robust, implying that CSD manifests prior to tipping.

Noise, network connectivity and return rates to the steady state collectively characterize the behavior of temporal summary statistics for the two-patch model studied here. Assuming spatial homogeneity, we showed that increasing the level of coupling between patches reduces signal strengths by decreasing their magnitude relative to those obtained for isolated populations. The lag-1 autocorrelation function exhibits non-monotonic behavior with increasing coupling strength. The simulation study shows that the coefficient of variation is the most robust temporal indicator across different coupling regimes, as well as for various environmental conditions. These predictions for the behavior of the leading indicators are robust even if the constraint of spatial homogeneity is relaxed. The analytic expressions derived in the homogeneous case are useful for prediction in spatially heterogeneous systems, where having patch-specific indicators that account for coupling between subsystems becomes more important.

Increasing the degree of coupling induces synchronous dynamics in both patches in the heterogeneous model. When a good-quality patch is available, rescue effects due to dispersal buffer the system from a catastrophic collapse by introducing synchrony in the network dynamics. Alternatively, in the heterogeneous system with a weak source patch, the dynamics of the poor quality patch follow those of the declining subpopulation over a short transient, and both subpopulations simultaneously decrease toward extinction. These results suggest that both patches in the system should be monitored. However, we have a stronger result in the scenario of observing a tipping cascade. In particular, a signal in one patch is sufficient to inform our understanding of its counterpart. This finding can have important implications in several settings. For example, nearly all species are buffeted by stochasticity and many of them could have Allee dynamics (Liebhold and Bascompte 2003).

A key shortcoming of our findings relates to the limits of applicability of CSD, which forms the basis of research on early warning signals (Clements and Ozgul 2018). As is typical of the transition from theory to practice, we must be careful in applying our methods, rooted in alternative stable states theory and more specifically bifurcation theory, to real ecological data (Burthe et al. 2016). Moreover, while critical slowing down and its associated components of instability are present in some biological models, it is absent from several others, including systems displaying catastrophic failures (Boerlijst et al. 2013). We must also be cognizant of the fact that predictability does not necessarily allow for prevention, as the fate of the system may be unavoidable (Boettiger et al. 2013).

In addition, we used a simple model for a saddle-node bifurcation to describe the intrinsic patch dynamics, but the framework can be generalized for bistable ecosystems present in nature. For instance, accounting for spatial structure and movement pathways through networks (Suweis and D’Odorico 2014) is a natural way to extend the two-patch model presented here. Because we are interested in the scenario of noise-induced tipping via a saddle-node bifurcation, with a smooth and gradual approach to the tipping point, extinction is highly likely. A factor that may influence the time to extinction is the dispersal rate between patches, which could lead to a rescue effect. In other words,we emphasize the importance of transient dynamics in our analyses over a finite time horizon and do not explore persistence in the long-term dynamics of the system. We do note, however, that prior work explored the role of patch dispersion in the persistence of stochastic populations, through a linear model without Allee effects (Evans et al. 2013) and a density-dependent logistic model without Allee effects (Hening et al. 2018). In summary, we have studied a general model for early warning systems of tipping cascades. Although the analytic expressions for leading indicators need to be numerically evaluated for the spatially heterogeneous model, they still play a key role in the identification of critical slowing down in coupled ecological networks.
